# Supplementary figures and images for: Association of potential salivary biomarkers with diabetic retinopathy and its severity in type-2 diabetes mellitus: a proteomic analysis by mass spectrometry
Source: PeerJ. 2016 May 12;4:e2022. doi: 10.7717/peerj.2022 (PMC4893325; doi:10.7717/peerj.2022)

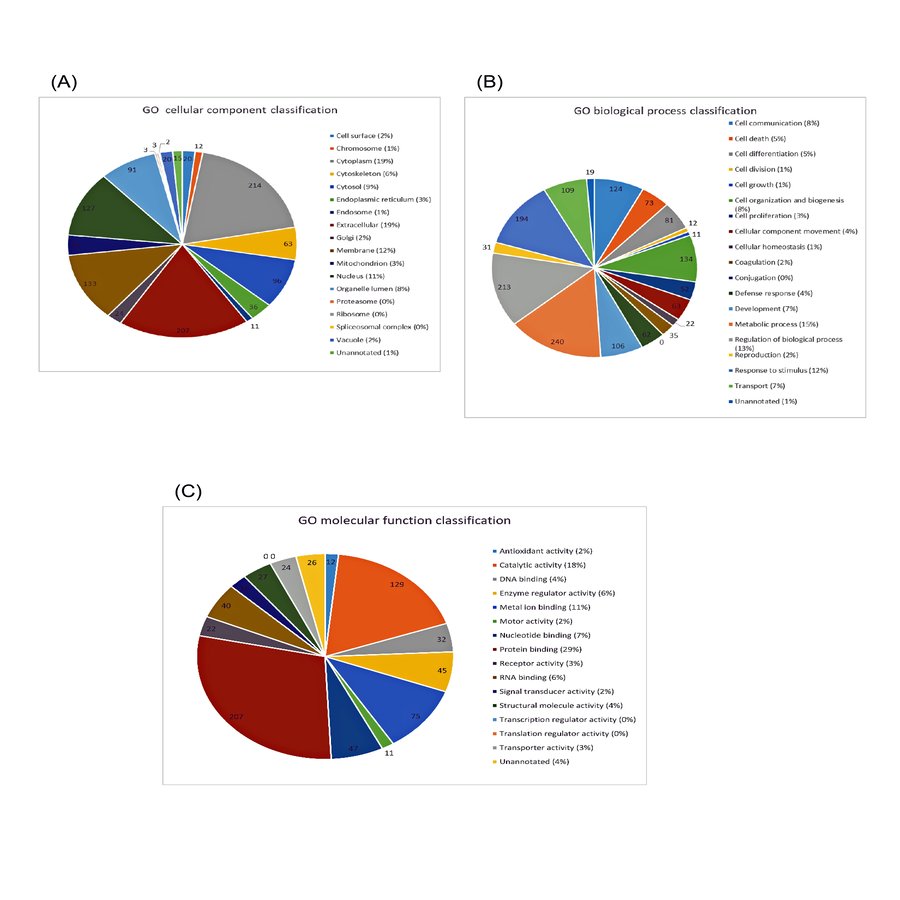

Supplement: Supplemental Information 2 — The proteins were classified based on (A) subcellular localization, (B) biological processes involved and (C) molecular functions. [file peerj-04-2022-s002.jpg]

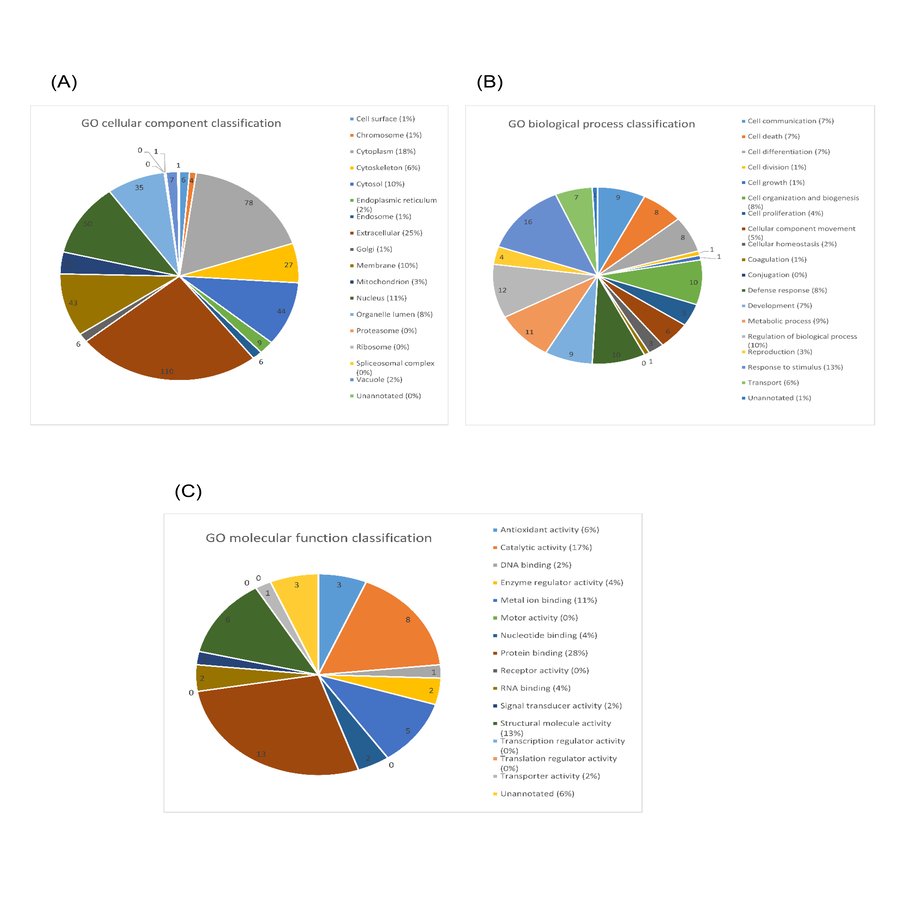

Supplement: Supplemental Information 3 — The proteins were classified based on (A) subcellular localization, (B) biological processes involved and (C) molecular functions. [file peerj-04-2022-s003.jpg]

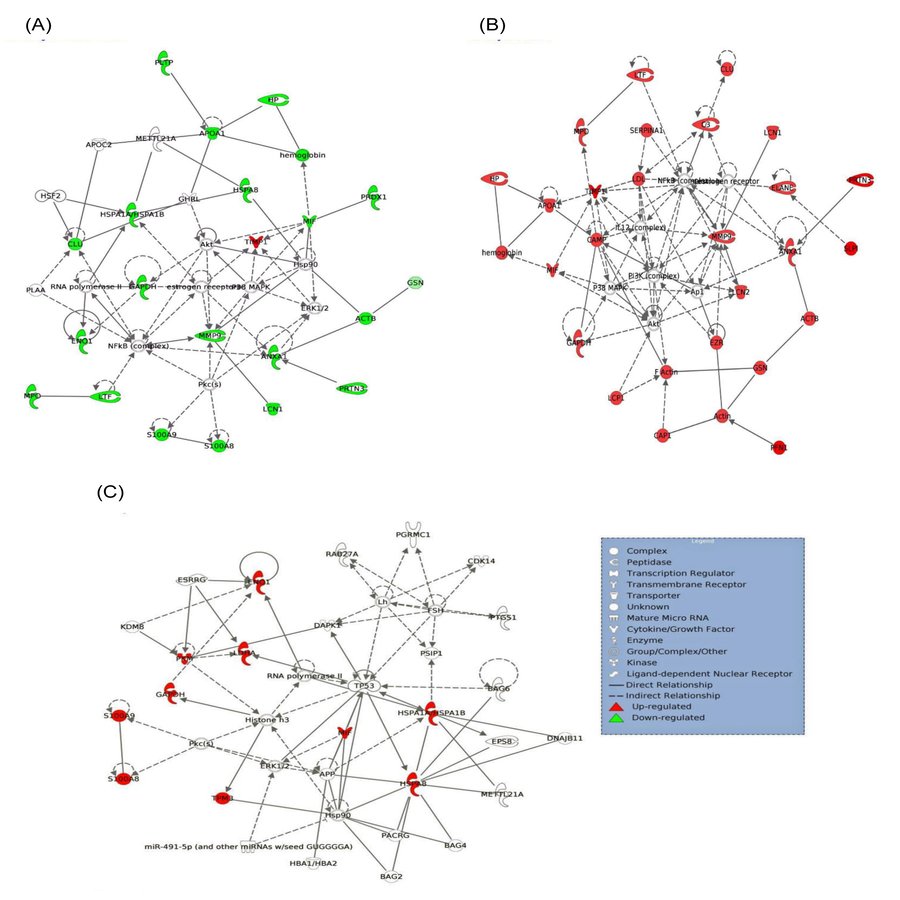

Supplement: Supplemental Information 4 — Network (A) contains 35 proteins involved in connective tissue disorders, immunological disease and inflammatory disease in which 22 proteins were identified from the NPDR disease group. Network (B) contains 35 proteins involved in cellular movement, hematological system development and function and immune cell trafficking in which 28 proteins are identified from the PDR disease group. Network (C) contains 26 proteins involved in cellular growth and proliferation, cancer and carbohydrate metabolism in which 10 proteins are identified in PDR disease group. (ACTB = actin, cytoplasmic 1, ANXA1 = annexin A1, APOA1 = apolipoprotein A-I, CAMP = cathelicidin antimicrobial peptide, CAP1 = adenylyl cyclase-associated protein 1, CLU = clusterin, C3 = complement C3, ELANE = neutrophil elastase, ENO1 = alpha-enolase isoform 1, EZR = ezrin, GAPDH = glyceraldehyde-3-phosphate dehydrogenase isoform 2, GSN = gelsolin isoform d, HBA1/HBA2 = hemoglobin subunit alpha, HP = haptoglobin isoform 2, HSPA8 = heat shock cognate 71 kDa protein isoform 1, HSPA1A/HSPA1B = heat shock 70 kDa protein 1A/1B, LCP1 = plastin-2, LCN1 = lipocalin-1 isoform 1, LCN2 = neutrophil gelatinase-associated lipocalin, LDHA = L-lactate dehydrogenase A chain isoform 3, LTF = lactotransferrin isoform 1, MIF = macrophage migration inhibitory factor, MMP9 = matrix metalloproteinase-9, MPO = myeloperoxidase, PKM = pyruvate kinase isozymes M1/M2 isoform c, PLTP = phospholipid transfer protein isoform a, PRDX1 = peroxiredoxin-1, PRTN3 = profilin-1, SERPINA1 = alpha-1-antitrypsin, SLPI = antileukoproteinase, S100A8 = protein S100-A8, S100A9 = protein S100-A9, TIMP1 = metalloproteinase inhibitor 1, TPM3 = tropomyosin alpha-3 chain isoform 2). [file peerj-04-2022-s004.jpg]
