# Supplementary material for: Association of potential salivary biomarkers with diabetic retinopathy and its severity in type-2 diabetes mellitus: a proteomic analysis by mass spectrometry
Source: PeerJ. 2016 May 12;4:e2022. doi: 10.7717/peerj.2022 (PMC4893325; doi:10.7717/peerj.2022)
Supplement: Supplemental Information 5 [file peerj-04-2022-s005.docx]

**S1 table.**  **Salivary proteins that are differentially expressed in PDR disease group compared to XDR disease group.**

| **Accession** | **Protein Name** | **Unique Peptide** | **Peptide (95%)** | **Coverage (%)** | **Fold change** |
| --- | --- | --- | --- | --- | --- |
| **Up-regulated** | | | | | |
| 355594753 | Clusterin preproprotein  [*Homo sapiens*] | 4 | 4 | 31.18 | 70.804 |
| 186910296 | Haptoglobin isoform 2 preproprotein [*Homo sapiens*] | 7 | 11 | 27.09 | 61.138 |
| 7706635 | Cornulin [*Homo sapiens*] | 4 | 17 | 54.34 | 55.219 |
| 342307069 | BPI fold-containing family A member 1 precursor [*Homo sapiens*] | 4 | 5 | 26.95 | 55.112 |
| 300244560 | Cysteine-rich secretory protein 3 isoform 1 precursor [*Homo sapiens*] | 2 | 7 | 24.81 | 52.793 |
| 4505185 | Macrophage migration inhibitory factor [*Homo sapiens*] | 1 | 2 | 18.26 | 52.749 |
| 24119203 | Tropomyosin alpha-3 chain isoform 2 [*Homo sapiens*] | 2 | 10 | 36.29 | 52.220 |
| 23238211 | Actin-related protein 2/3 complex subunit 2 [*Homo sapiens*] | 1 | 6 | 23.33 | 51.538 |
| 45592961 | BPI fold-containing family A member 2 precursor [*Homo sapiens*] | 8 | 8 | 32.93 | 51.432 |
| 222418647 | mucin-7 precursor [*Homo sapiens*] | 2 | 5 | 16.98 | 50.129 |
| 4506773 | protein S100-A9 [*Homo sapiens*] | 6 | 10 | 71.93 | 48.980 |
| 11321561 | Hemopexin precursor [*Homo sapiens*] | 3 | 14 | 39.83 | 48.782 |
| 4557014 | Catalase [*Homo sapiens*] | 2 | 17 | 47.63 | 48.407 |
| 4503107 | Cystatin-C precursor *[Homo sapiens*] | 4 | 4 | 59.59 | 46.785 |
| 4885063 | Fructose-bisphosphate aldolase C [*Homo sapiens*] | 1 | 6 | 28.30 | 45.572 |
| 4502101 | Annexin A1 [*Homo sapiens*] | 8 | 11 | 39.02 | 45.282 |
| 5729877 | Heat shock cognate 71 kDa protein isoform 1 [*Homo sapiens*] | 4 | 4 | 34.37 | 44.759 |
| 74271845 | Alpha-2-macroglobulin-like protein 1 precursor [*Homo sapiens*] | 9 | 26 | 23.45 | 44.309 |
| 15055535 | BPI fold-containing family B member 2 precursor [*Homo sapiens*] | 7 | 11 | 31.22 | 43.009 |
| 189163540 | Alpha-1-antitrypsin precursor [*Homo sapiens*] | 2 | 6 | 20.57 | 37.752 |
| 38455402 | Neutrophil gelatinase-associated lipocalin precursor [*Homo sapiens*] | 5 | 8 | 51.01 | 32.629 |
| 74272287 | Matrix metalloproteinase-9 preproprotein [*Homo sapiens*] | 4 | 20 | 38.47 | 31.557 |
| 4507509 | Metalloproteinase inhibitor 1 precursor [*Homo sapiens*] | 2 | 6 | 24.64 | 29.385 |
| 4503549 | Neutrophil elastase preproprotein [*Homo sapiens*] | 4 | 4 | 57.30 | 28.739 |
| 5031863 | Galectin-3-binding protein precursor [*Homo sapiens*] | 5 | 16 | 33.68 | 22.698 |
| 21614544 | Protein S100-A8 [*Homo sapiens*] | 5 | 5 | 36.56 | 22.059 |
