# Supplementary material for: Association of potential salivary biomarkers with diabetic retinopathy and its severity in type-2 diabetes mellitus: a proteomic analysis by mass spectrometry
Source: PeerJ. 2016 May 12;4:e2022. doi: 10.7717/peerj.2022 (PMC4893325; doi:10.7717/peerj.2022)
Supplement: Supplemental Information 6 [file peerj-04-2022-s006.docx]

**S2 table.** **Salivary proteins that are differentially expressed in NPDR patient group compared to XDR patient group.**

| **Accession** | **Protein Name** | **Unique peptides** | **Peptide (95%)** | **Coverage %** | **Fold change** |
| --- | --- | --- | --- | --- | --- |
| **Up-regulated** | | | | | |
| 4507509 | Metalloproteinase inhibitor 1 precursor [*Homo sapiens*] | 2 | 6 | 24.64 | 3.821 |
| **Down-regulated** | | | | | |
| 194272142 | Unconventional myosin-IXb isoform 2 [*Homo sapiens*] | 1 | 40 | 22.45 | 0.228 |
| 32455264 | Peroxiredoxin-1 [*Homo sapiens*] | 3 | 5 | 26.13 | 0.169 |
