# Supplementary material for: Association of potential salivary biomarkers with diabetic retinopathy and its severity in type-2 diabetes mellitus: a proteomic analysis by mass spectrometry
Source: PeerJ. 2016 May 12;4:e2022. doi: 10.7717/peerj.2022 (PMC4893325; doi:10.7717/peerj.2022)
Supplement: Supplemental Information 7 [file peerj-04-2022-s007.docx]

**S3 table The differentially expressed proteins identified from the study that interact with each other in the protein-protein network analysis.**

| **Protein Name** | **GO Biological process** | **GO Molecular function** | **Canonical pathways** | **Predicted interacting partner** |
| --- | --- | --- | --- | --- |
|  |  |  |  |  |
| Clusterin preproprotein [*Homo sapiens*] | cell organization and biogenesis; cell communication; cell death; regulation of biological process; response to stimulus; transport; metabolic process; coagulation; cell proliferation; development; cell differentiation; defense response | protein binding; catalytic activity | LXR/RXR activation, Clathrin-mediated endocytosis signalling, Atherosclerosis signaling, Production of Nitric Oxide and Reactive Oxygen Species in Macrophages, FXR/RXR activation,  IL-12 signaling and production in macrophages |  |
| Haptoglobin isoform 2 preproprotein [*Homo sapiens*] | metabolic process; cellular homeostasis; defense response; response to stimulus; cell death; regulation of biological process | catalytic activity; protein binding; antioxidant activity | IL-12 signaling and production in Macrophages, Acute phase response signaling | apolipoprotein A-I (direct)  hemoglobin (direct) |
| Macrophage migration inhibitory factor [*Homo sapiens*] | metabolic process; regulation of biological process; cell death; defense response; response to stimulus; cell communication; cell proliferation; transport; cell organization and biogenesis | catalytic activity; protein binding |  | hemoglobin (indirect)  peroxiredoxin-1  (direct)  metalloproteinase inhibitor 1  (direct) |
| Tropomyosin alpha-3 chain isoform 2 [*Homo sapiens*] | cellular component movement; regulation of biological process; cell communication; response to stimulus | protein binding; structural molecule activity |  |  |
| Protein S100-A9 [*Homo sapiens*] | cellular component movement; defense response; response to stimulus; metabolic process; cell death; regulation of biological process; cell communication; cell growth; cell organization and biogenesis; cellular homeostasis | signal transducer activity; metal ion binding; protein binding; antioxidant activity; catalytic activity | Role of IL-17A in Psoriasis | protein S100-A8  (direct) |
| Annexin A1 [*Homo sapiens*] | defense response; regulation of biological process; response to stimulus; cellular component movement; cell communication; metabolic process; transport; cell differentiation; development; cell organization and biogenesis; cell death; cell proliferation; reproduction | DNA binding; RNA binding; enzyme regulator activity; protein binding; structural molecule activity; metal ion binding |  | actin, cytoplasmic 1 (direct)  myeloblastin (direct) |
| Heat shock cognate 71 kDa protein isoform 1 *[Homo sapiens*] | cell organization and biogenesis; metabolic process; regulation of biological process; transport; response to stimulus; cell communication; cell differentiation; cellular component movement; development | nucleotide binding; protein binding; catalytic activity; RNA binding | Clathrin-mediated endocytosis signaling | heat shock 70 kDa protein 1A/1B  (direct) |
| Alpha-enolase isoform 1 [*Homo sapiens*] | metabolic process; regulation of biological process; response to stimulus; cell growth; cell organization and biogenesis | metal ion binding; DNA binding; catalytic activity; protein binding; RNA binding | Glycolysis I, Gluconeogenesis I | glyceraldehyde-3-phosphate dehydrogenase isoform 2 (indirect) |
| Myeloblastin precursor [*Homo sapiens*] | metabolic process; cell proliferation; regulation of biological process; cell organization and biogenesis; transport; cell differentiation; development | catalytic activity; protein binding |  | annexin A1 (direct) |
| Profilin-1 [*Homo sapiens*] | development; transport; metabolic process; regulation of biological process; cell organization and biogenesis; coagulation; response to stimulus; cell death; cellular component movement | enzyme regulator activity; nucleotide binding; protein binding; RNA binding | Regulation of actin-based motility by Rho, Actin cytoskeleton signaling | Actin (direct) |
| Myeloperoxidase precursor [*Homo sapiens*] | response to stimulus; metabolic process; defense response; cell organization and biogenesis; regulation of biological process; cell death | antioxidant activity; catalytic activity; metal ion binding | Production of Nitric Oxide and Reactive Oxygen Species in Macrophages | lactotransferrin isoform 1 (direct)  cathelicidin antimicrobial peptide (indirect) |
| Alpha-1-antitrypsin precursor [*Homo sapiens*] | response to stimulus; transport; defense response; coagulation; metabolic process; regulation of biological process | protein binding; enzyme regulator activity | LXR/RXR activation, Clathrin-mediated endocytosis signalling, Atherosclerosis signalling, Production of Nitric Oxide and Reactive Oxygen Species in Macrophages,  FXR/RXR activation, IL-12 signaling and production in Macrophages,  Acute phase response signaling | Low density lipoprotein (direct) |
| Cathelicidin antimicrobial peptide preproprotein [*Homo sapiens*] | metabolic process; regulation of biological process; defense response; response to stimulus; cell proliferation; development; cell organization and biogenesis | protein binding |  | Myeloperoxidase (indirect)  glyceraldehyde-3-phosphate dehydrogenase isoform 2 (direct) |
| Heat shock 70 kDa protein 1A/1B [*Homo sapiens*] | nucleotide binding; receptor activity; RNA binding; protein binding; catalytic activity | cell organization and biogenesis; metabolic process; response to stimulus; cell proliferation; regulation of biological process; cell death; cell growth; cell differentiation; development; cell communication |  | heat shock cognate 71 kDa protein isoform 1 (direct) |
| Complement C3 precursor [*Homo sapiens*] | defense response; regulation of biological process; response to stimulus; metabolic process; cell communication; transport; development; cell organization and biogenesis | enzyme regulator activity; protein binding | LXR/RXR activation, FXR/RXR activation, Acute phase response signaling | Low density lipoprotein (indirect) |
| L-lactate dehydrogenase A chain isoform 3 [*Homo sapiens*] | metabolic process; development; cell communication; response to stimulus | nucleotide binding; catalytic activity; protein binding |  | pyruvate kinase isozymes M1/M2 isoform c (indirect) |
| Antileukoproteinase precursor [*Homo sapiens*] | metabolic process; regulation of biological process | enzyme regulator activity; protein binding |  | neutrophil elastase (indirect) |
| Ezrin [*Homo sapiens*] | cell organization and biogenesis; cell differentiation; cellular component movement; development; response to stimulus; regulation of biological process; metabolic process; transport | protein binding; RNA binding | Leukocyte extravasation signaling, Actin cytoskeleton signaling | Actin (direct) |
| Neutrophil gelatinase-associated lipocalin precursor [*Homo sapiens*] | transport; cell death; response to stimulus; metabolic process; regulation of biological process; cellular homeostasis; cell organization and biogenesis; cell communication; defense response | protein binding; transporter activity; metal ion binding |  | matrix metalloproteinase-9 (direct) |
| Matrix metalloproteinase-9 preproprotein [*Homo sapiens*] | development; response to stimulus; metabolic process; reproduction; cell growth; cell organization and biogenesis; cell differentiation; cell communication; regulation of biological process; cell death; cellular component movement; transport | protein binding; catalytic activity; metal ion binding | LXR/RXR activation, Atherosclerosis signalling, Leukocyte extravasation signaling | neutrophil gelatinase-associated lipocalin (direct) |
| Lactotransferrin isoform 1 precursor [*Homo sapiens*] | regulation of biological process; defense response; response to stimulus; metabolic process; transport; cellular homeostasis; cell communication; cell proliferation; cell death; cell differentiation; development; cell organization and biogenesis | DNA binding; catalytic activity; metal ion binding; protein binding; enzyme regulator activity |  | Myeloperoxidase (direct) |
| Metalloproteinase inhibitor 1 precursor [*Homo sapiens*] | transport; coagulation; response to stimulus; cell proliferation; regulation of biological process; metabolic process; cell organization and biogenesis; cell death; cell differentiation; development; cellular component movement |  | Leukocyte extravasation signaling | macrophage migration inhibitory factor (indirect) |
| Pyruvate kinase isozymes M1/M2 isoform c [*Homo sapiens]* | response to stimulus; development; metabolic process; cell death | metal ion binding; catalytic activity; protein binding; nucleotide binding; RNA binding | Glycolysis I, | L-lactate dehydrogenase A chain isoform 3 (indirect) |
| Neutrophil elastase preproprotein [*Homo sapiens]* | metabolic process; regulation of biological process; response to stimulus; defense response; cellular homeostasis; transport; cell organization and biogenesis; cell communication; cell proliferation; cellular component movement | protein binding; catalytic activity |  | Antileukoproteinase (indirect) |
| Apolipoprotein A-I preproprotein [*Homo sapiens]* | metabolic process; regulation of biological process; cell proliferation; transport; response to stimulus; cell communication; coagulation; cell organization and biogenesis; cell differentiation; development; cellular component movement; defense response | transporter activity; protein binding; enzyme regulator activity | LXR/RXR activation, Clathrin-mediated endocytosis signalling, Atherosclerosis signalling, Production of Nitric Oxide and Reactive Oxygen Species in Macrophages, FXR/RXR activation, IL-12 signaling and production in macrophages, Acute phase response signaling | phospholipid transfer protein isoform a (direct)  haptoglobin isoform 2 (direct) |
| Adenylyl cyclase-associated protein 1 [*Homo sapiens*] | cell organization and biogenesis; cellular component movement; transport; cell communication; regulation of biological process; response to stimulus; metabolic process; cell differentiation; development; coagulation | protein binding |  | F actin (indirect)  Actin (direct) |
| Actin, cytoplasmic 1 [*Homo sapiens]* | cell organization and biogenesis; metabolic process; cellular component movement; cell differentiation; development; response to stimulus; coagulation; cell communication; regulation of biological process; transport; defense response | nucleotide binding; DNA binding; structural molecule activity; protein binding | Clathrin-mediated endocytosis signalling, Leukocyte extravasation signaling, Regulation of actin-based motility by Rho, Actin cytoskeleton signaling | Annexin A1 (direct)  gelsolin isoform d (direct) |
| Gelsolin isoform d [*Homo sapiens]* | cell death; cell organization and biogenesis; cell differentiation; development; transport; regulation of biological process; response to stimulus; cell communication | protein binding; metal ion binding | Regulation of actin-based motility by Rho, Actin cytoskeleton signaling | actin, cytoplasmic 1 (direct)  F-actin (direct)  actin (direct) |
| Plastin-2 [*Homo sapiens*] | response to stimulus; cell communication; regulation of biological process; cellular component movement; development; transport; cell organization and biogenesis | protein binding; metal ion binding |  | F-actin (indirect) |
| Glyceraldehyde-3-phosphate dehydrogenase isoform 2 [*Homo sapiens*] | cell organization and biogenesis; metabolic process; regulation of biological process; cell death; defense response; response to stimulus | catalytic activity; protein binding; nucleotide binding | Glycolysis I, Gluconeogenesis I | alpha-enolase isoform 1 (indirect) |
| Phospholipid transfer protein isoform a precursor [*Homo sapiens*] | metabolic process; transport; cellular component movement |  | LXR/RXR activation, FXR/RXR activation | apolipoprotein A-I (direct) |
| Protein S100-A8 [*Homo sapiens]* | cellular component movement; defense response; response to stimulus; metabolic process; cell death; regulation of biological process; cell growth; cell organization and biogenesis; cellular homeostasis; cell communication | metal ion binding; protein binding | LXR/RXR activation, Clathrin-mediated endocytosis signalling, Atherosclerosis signalling, Production of Nitric Oxide and Reactive Oxygen Species in Macrophages, Role of IL-17A in Psoriasis, IL-12 signaling and production in Macrophages | protein S100-A9 (direct) |
| Lipocalin-1 isoform 1 precursor [*Homo sapiens*] | metabolic process; transport; regulation of biological process; response to stimulus | enzyme regulator activity |  | matrix metalloproteinase-9 (direct) |
| Hemoglobin subunit alpha [*Homo sapiens*] | transport; cell death; regulation of biological process; response to stimulus; metabolic process; cell organization and biogenesis | antioxidant activity; catalytic activity; transporter activity; metal ion binding; protein binding |  | macrophage migration inhibitory factor (indirect)  apolipoprotein A-I (direct)  haptoglobin isoform 2 (direct) |
| Peroxiredoxin-1 [*Homo sapiens]* | response to stimulus; development; cell proliferation; metabolic process; cell communication; regulation of biological process; defense response; transport | antioxidant activity; catalytic activity; protein binding; RNA binding |  | macrophage migration inhibitory factor (direct) |
